# The Feasibility of Prenatal Ultrasonic Screening for Developmental Dysplasia of the Hip

**DOI:** 10.3390/diseases12080188

**Published:** 2024-08-17

**Authors:** Claudia Bevilacqua, Virginia Boscarato, Giovanni Pieroni, Eva Fraternali, Giuliano Lattanzi, Simone Domenico Aspriello, Antonio Pompilio Gigante, Alessandro Cecchi

**Affiliations:** 1 Adult and Pediatric Orthopedic Clinic, Azienda Ospedaliero-Universitaria Ospedali Riuniti di Ancona, Via Conca 71, 60126 Torrette, Italy; claudia.bevilaqua@ospedaliriuniti.marche.it; 2 Second-Level Prenatal Diagnosis, Ospedale di Comunità di Loreto, AST Ancona, Via F. D’Assisi 1, 60025 Loreto, Italy; virginia.boscarato@sanita.marche.it (V.B.); alessandro.cecchi@sanita.marche.it (A.C.); 3 Radiological Sciences, SOD Radiologia Materno Infantile, Azienda Ospedaliero-Universitaria Ospedali Riuniti di Ancona, Via F. Corridoni 11, 60123 Ancona, Italy; giovanni.pieroni@ospedaliriuniti.marche.it; 4 Adult and Pediatric Orthopedic Clinic, Dip. Scienze Cliniche e Molecolari, Università Politecnica delle Marche, Ancona, Via Conca 71, 60126 Torrette, Italy; e.fraternali@pm.univpm.it (E.F.); giuliano.lattanzi@gmail.com (G.L.); a.p.gigante@staff.univpm.it (A.P.G.); 5 ASD Dental Clinic, 61121 Pesaro, Italy; 6 INRCA, ICRS, via della Montagnola, 60127 Ancona, Italy

**Keywords:** developmental dysplasia of the hip, DDH, congenital dislocation of the hip, prenatal ultrasound screening of the hip, Graf technique, alpha angle, beta angle, newborn hip screening

## Abstract

Background: developmental dysplasia of the hip (DDH) is a condition characterized by abnormal hip development in infancy. Early diagnosis allows for effective treatment, while late presentation often necessitates complex surgical interventions. Current recommendations advise screening between the 6th and 8th week postnatal using an ultrasound, typically employing the Graf method. However, there is no universal consensus on whether ultrasound screening significantly increases treatment likelihood compared to clinical examination-guided ultrasound. This study aims to explore the feasibility of prenatal ultrasound for the early identification of DDH risk. Methods: This prospective observational study involved 100 pregnant women undergoing fetal hip ultrasounds during the second and third trimesters. Using the modified Graf method, alpha and beta angles were calculated on the fetus. Postnatally, alpha and beta angles were compared with the prenatal values. Results: Prenatal ultrasound at the 24th week showed inconclusive results because of the difficulty in identification of Graf landmarks, while ultrasound at the 34th week proved to be a reliable and safe method for the quantitative determination of alpha and beta angles. Significant correlations were found between prenatal and postnatal alpha and beta angles. Moreover, significant differences in prenatal alpha and beta values were observed in patients developing mature/immature hips postnatally. Conclusions: Prenatal diagnostics show promise for predicting infant hip development. Further research is warranted to validate correlation strength and clinical applicability.

## 1. Introduction

Developmental dysplasia of the hip (DDH), also referred to as congenital dislocation of the hip, encompasses a spectrum of pathological conditions characterized by abnormal hip development in infancy. This can range from complete dislocation to bone immaturity with a stable hip [1]. 

Early diagnosis of DDH plays a pivotal role in ensuring effective and straightforward treatment, fostering high family compliance and positive outcomes [2]. 

However, cases that present later often require intricate surgical interventions [2,3].

Current protocols involve clinical examinations of newborns to assess hip stability, with the goal of preventing the need for delayed treatments linked to long-term hip deformities, gait disorders, and arthritis.

The introduction of ultrasound technology for assessing infant hip pathology stands out as a significant advancement over the past three decades. This technique allows for precise visualization of all components, both mineralized and non-mineralized, of the infant hip, facilitating the early detection of joint abnormalities. Key ultrasound assessment techniques for DDH include Graf’s method [4,5], Harcke’s method [6], and Morin–Terjesen’s method [7,8].

At our center, the method used is according to Graf. The Graf method presupposes the recognition of the lateral standard sector. On this scan, the inclination angle of the osseous acetabular roof (alpha angle) and the inclination of the cartilaginous acetabular roof (beta angle) are determined. The alpha angle determines the type, and the beta angle determines the subtype in the eponymous classification (Table 1).

To standardize the acquisition, ultrasound landmarks necessary for overcoming usability checklists must be identified, without which it is not possible to proceed with the measurement [4,5,9].

Despite the widespread use of neonatal screening protocols (combining clinical and ultrasound assessments for DDH), there remains insufficient evidence to definitively highlight the advantages and optimal timing for such examinations. A 2011 Cochrane review reported inconsistent evidence regarding universal ultrasound screening compared to targeted ultrasound or clinical examination alone. No ultrasound screening strategy has demonstrated enhanced clinical outcomes, including cases of late-diagnosed DDH requiring surgery [10].

The early identification of patients at risk for the development of DDH could help better focus diagnostic–therapeutic attention, reducing the effort of universal screening. 

The aim of this study was to verify the feasibility of diagnosing developmental dysplasia of the hip (DDH) using prenatal ultrasound during the routine gynecological check-ups of the fetus in the second and third trimesters of pregnancy.

## 2. Materials and Methods

This prospective observational study was conducted at the Prenatal Diagnosis Center at the Loreto Community Hospital (Italy), the Pediatric Orthopedic Clinic Department, and the Pediatric Radiology Department at Salesi Hospital in Ancona (Italy), from January 2023 to March 2024.

Informed consent was obtained from all women and caregivers of the infants involved in the study. This study adheres to the principles outlined in the Helsinki Declaration.

A total of 100 pregnant women were recruited from the Prenatal Diagnosis Center to undergo scheduled obstetric ultrasound examinations during the second and third trimesters.

Inclusion criteria were as follows:

- Women aged 18 to 45 years;
- Physiological pregnancies;
- Pregnancies at term;
- Complete follow-up through 18 weeks postpartum.

Exclusion criteria were as follows:

- Twin or multiple pregnancies;
- Suspected or diagnosed fetal abnormalities;
- Mother’s pathological obesity (BMI > 30 kg/m^2^).

In addition to the routine examination of the fetus scheduled for the gestational week (at 24th and 34th), an ultrasonographic examination of the hips was performed. The examinations were conducted by a single expert obstetrician–gynecologist. The objective of the investigation was to acquire a neutral lateral scan of the fetal hip joint, on which to measure the alpha and beta angles by adapting the method described by Graf for infants.

GE HealthCare Voluson E10 with a convex probe C2-9 (3–9 mHz) was used for the measurement. It should be noted that in most cases, it was possible to measure only one hip, as the technique allowed for the analysis of only the hip presented towards the probe. Some maneuvers, such as changing the mother’s position or manipulating with the probe can be used to facilitate the presentation of the hip. In 10 cases, it was not possible to collect a standard scan of the fetus’s hip, so they were excluded from the evaluation and the results were derived from a total of 90 cases.

**Figure 1:**
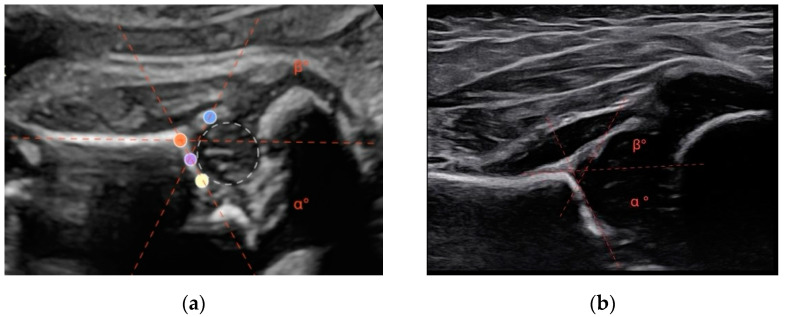
(**a**) During the ultrasonographic examination of a 34-week-old fetus, the hip near the maternal abdominal wall was visualized. The acetabular roof (orange), the turning point (purple), the lower edge of the ossified ilium (yellow), and the labrum (blue). The angles alpha and beta are determined by the intersection of the lines connecting the specified reference points. In this case, the alpha angle measures 61°, while the beta angle measures 64°. (**b**) The same patient, in the postnatal ultrasound at 6 weeks of age, shows an alpha angle of 65° and a beta angle of 60°.

A modified Graf method was performed. This involves acquiring a lateral scan of the newborn’s hip. Similarly to the Graf method for infants, the alpha angle is defined as the angle formed between a line tangent to the iliac wing and a line tangent to the bony roof. The beta angle is formed by the line tangent to the iliac wing and a line tangent to the cartilaginous roof [9,11] (Figure 1).

As in the routine neonatal hip screening program, infants underwent an ultrasound examination 6 weeks after birth, according to the Graf method. The ultrasound images were obtained by a GE HealthCare Logiq device with a linear ML6–15 Mhz probe. The examination was performed by a single independent radiologist, separate from the physician who conducted the prenatal investigations. The values of the alpha and beta angles were determined, and based on these, the hips were classified according to the Graf classification (types Ia, Ib, IIa, IIb, IIc, IId, IIIa, IIIb, and IV). For statistical purposes, patients were divided into two subgroups: group A, including type I hips; and group B, including type II, III, and IV hips.

Statistical analysis was performed to verify the differences between the alpha and beta values between group A and group B.

The correlation between prenatal and postnatal alpha and beta angle values was also analyzed in the entire population.

### Statistical Analysis

Statistical analysis of the data was performed using SPSS software version 29.0.2.0 (IBM SPSS Corp.; Armonk, NY, USA). The normal distribution of alpha and beta values was checked using the Shapiro–Wilk test. To identify if there was a statistically significant difference in prenatal alpha angles, the Mann–Whitney U test was performed. The Spearman correlation coefficient was calculated to assess the relationship between the alpha angle value (in degrees) at 34 weeks of gestation and the alpha angle value (in degrees) at 6 weeks postnatal. The same methodologies were applied to the beta angle (in degrees).

## 3. Results

The baseline characteristics of the two groups are summarized in Table 2.

In the second trimester, scans showed inconclusive results because, in our experience, it was not possible to assess with accuracy the ultrasonographic findings necessary for calculating the alpha and beta angles.

In the third trimester, scans allowed the measurement of alpha and beta angles in 90 out of 100 examinations. The primary obstacle to acquiring these scans was the failure to obtain a lateral view of the hip due to the fetus’s positioning.

Using the Shapiro–Wilk test, data from the 3rd trimester did not have a normal distribution. The Shapiro–Wilk test statistic for prenatal alpha angle values was W = 0.977, with a *p*-value = 0.104. The Shapiro–Wilk test statistic for prenatal beta angle values was W = 0.957, with a *p*-value = 0.005. The Shapiro–Wilk test statistic for postnatal alpha angle values was W = 0.933, with a *p*-value < 0.001. The Shapiro–Wilk test statistic for postnatal beta angle values was W = 0.907, with a *p*-value < 0.001. Since the *p*-values are less than 0.05, the data do not appear to be normally distributed.

Comparing prenatal alpha angle values between Group A and Group B using the Mann–Whitney test revealed a statistically significant difference between the two groups (U = 362.000, *p* < 0.001). Comparing prenatal beta angle values between Group A and Group B using the Mann–Whitney test also showed a statistically significant difference between the two groups (U = 1763.500, *p* < 0.001). Comparing postnatal alpha angle values between Group A and Group B using the Mann–Whitney test revealed a statistically significant difference between the two groups (U = 0.000, *p* < 0.001). Comparing postnatal beta angle values between Group A and Group B using the Mann–Whitney test also showed a statistically significant difference between the two groups (U = 1804.500, *p* < 0.001).

**Figure 2:**
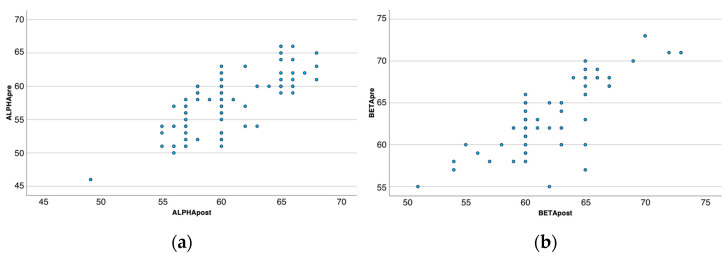
(**a**) Scatter plot prenatal/postnatal α-angles (**b**). Scatter plot prenatal/postnatal β-angles.

The variables (prenatal alpha/postnatal alpha and prenatal beta/postnatal beta) exhibit a positive monotonic relationship, as evidenced by scatterplots (Figure 2).

Spearman rank correlation analyses revealed that the prenatal alpha angle value has a strong correlation with the postnatal alpha angle, rho = 0.717, *p* < 0.001, and the prenatal beta angle has a strong correlation with the postnatal beta angle, rho = 0.737, *p* < 0.001.

## 4. Discussion

Developmental dysplasia of the hip (DDH) is among the most frequent developmental anomalies in newborns, potentially leading to significant long-term disabilities. Given the relevant social and economic impacts, timely diagnosis of this condition is a goal that must be pursued not only in countries that apply universal screening but also in those that do not.

Ultrasound examinations of the fetus during the second and third trimesters are widely used in gynecological check-ups; nevertheless, commonly the fetal hip joint is not investigated, and few sonographic studies are available about the evolution of the neonatal hip from fetal development to the postnatal period.

Wagner et al. reported that between the 14th and 19th week of gestation, quantitative evaluation of bone structures is not possible, while from the 21st week, it is feasible to measure alpha and beta angles, which become more reliable as pregnancy progresses [12]. Stiegler et al. stated that the hip joint becomes sonographically mature at 34 weeks of gestation [13]. Baroti et al., by a combined morphological and ultrasound examination, confirmed that the alpha angle can be determined only after 18 weeks and that exact measures need a more advanced fetal development [14].

In a cohort study from the 37th week of gestation, Contro et al. demonstrated that the application of the Graf technique, currently used in postnatal ultrasound, can also be adapted to prenatal ultrasound with good reproducibility [15].

The results of this study are consistent with evidence from previous work in the literature. Our data indicated that ultrasound examination at the 24th week of pregnancy does not allow for accurate quantitative and qualitative assessment of alpha and beta values; conversely, ultrasonographic evaluation during the 34th week of gestation proved to be a reliable and safe method for the quantitative determination of alpha and beta angles.

Early ultrasound examination could potentially detect severe forms of DDH as dislocations, even if this condition generally develops in more advanced stages of fetal growth because the hip joint is less stable in perinatal life [14,16,17].

Contro et al. reported that, although there is no linear correlation between prenatal and postnatal values, the alpha angle systematically increases with development while the beta angle decreases [15].

In the study by Stiegler et al., which evaluated through sonography the fetal and newborn hip, the mean alpha angle found prenatally at 34 weeks was above the level of a mature hip joint and the values increased after birth, while there were no differences in the mean beta angles measured pre and postnatally [13].

Finally, Komut et al. in a study of 84 patients, observed a concordance between prenatal and postnatal alpha values [18].

In accordance with these authors, data from the present study showed a significant correlation at the 34th week of pregnancy (*p* < 0.001) between prenatal and postnatal alpha and beta values. Specifically, the alpha angle increased between the 34th week of gestation and the postnatal period, while the beta angle decreased with fetal development.

Our analyses also revealed a significant difference in alpha and beta angle values between infants with mature (Graf I) and immature hips (Graf II) both in prenatal and in postnatal examinations. Thus, data showed a strong correspondence in the maturity level of the hip between the fetuses at the late weeks of pregnancy and the newborns. This suggests that prenatal assessment can be used to identify patients at risk of hip immaturity. In our sample, there were no dislocated or decentered hips (Graf IV) in either the prenatal or postnatal findings, so no data are available on the ultrasonographic evaluation of hips in this situation. To our knowledge, no papers with these findings were produced.

A limitation of the present study was that it showed the results of the acquisition and analysis only of the hip closer to the mother’s abdominal wall. Further studies are planned, adopting various technical measures (changing fetal and maternal position, using as needed a linear, convex, or even transvaginal probe) to evaluate both fetal hips and reduce the risk of missed diagnoses of DDH.

## 5. Conclusions

Despite the cautions due to the limited sample size and the evaluation of only one hip for each fetus, the present study confirmed that prenatal ultrasonography represents a viable and safe evaluation method of the fetal hip joint. Data indicated that at the 34th week of gestation, it is possible to determine the alpha and beta angles, and that there is a significant correlation (*p* < 0.001) between prenatal and postnatal values.

The analysis of the results also revealed a significant difference in the measurements of the alpha and beta angles between mature hips (Graf I) and immature hips (Graf II), in both prenatal and postnatal examinations.

Morphological ultrasound screening during the third trimester of pregnancy could identify patients at risk for developing developmental dysplasia of the hip (DDH), facilitating the early identification of hips that require postnatal evaluation and treatment.

The present study lays the groundwork for potentially including hip evaluation in routine morphological ultrasonographic assessment of the fetus.

## Figures and Tables

**Table 1:** Graf’s ultrasound classification [4,5].

Type	α-Angle	β-Angle	Description	Bony Roof	Bony Rim	Cartilage Roof
Ia	≥60	≤55	Mature hip	Good	Blunt	Covers the f.h ^1^
Ib	>60	>55	Mature hip	Good	Blunt	Cover the f.h
IIa	50–59	>55	Physiologically immature (<3 mth)	Deficient	Rounded	Covers the f.h ^1^
IIb	50–59	<55	Delay of ossification (>3 mth)	Deficient	Rounded	Covers the f.h ^1^
IIc	43–49	<77	Critical hip	Severely deficient	Rounded to flattened	Still covers the f.h ^1^
IId	43–49	>77	Decentering hip	Severely deficient	Rounded to flattened	Displaced
III	<43	>77	Dislocated hip	Poor	Flattened	Labrum pressed upward
IV	<43		Dislocated hip	Poor	Flattened	Labrum pressed downward

^1^ Femoral head.

**Table 2:** General data of the patients. Group A = mature hips. Group B = immature hips.

	Group A	Group B
N° of patients	58 (64.4%)	32 (35.6%)
Graf classification		
Ia (%)	4 (6.9%)	
Ib (%)	54 (93.1%)	
IIa (%)		31 (96.9%)
IIb (%)		0
IIc (%)		1 (3.1%)
Gender		
Male *n* (%)	24 (41.4%)	10 (31.2%)
Female *n* (%)	34 (58.6%)	22 (68.8%)
Side		
Right	30 (51.7%)	12 (37.5%)
Left	28 (48.3%)	20 (62.5%)
Family history of DDH	9 (15.5%)	7 (21.9%)
Ethnicity		
Caucasian	46 (79.3%)	29 (90.6%)
African	9 (15.5%)	3 (9.4%)
Asian	3 (5.2%)	0
Multiparous	17 (29.3%)	8 (25%)
Primiparous	41 (70.7%)	24 (75%)

## Data Availability

The data presented in this study are available on request from the corresponding author. The data are not publicly available due to privacy.
